# High Risk for Attention-Deficit Hyperactive Disorder in Children with Strabismus: A Nationwide Cohort Study from the National Health Insurance Research Database

**DOI:** 10.3390/life11111139

**Published:** 2021-10-26

**Authors:** Chia-Ying Tsai, Chien-Chia Su, Yao-Lin Liu, I-Ju Tsai, Tzu-Hsun Tsai

**Affiliations:** 1 Department of Ophthalmology, Fu Jen Catholic University Hospital, Fu Jen Catholic University, New Taipei City 243, Taiwan; 140230@mail.fju.edu.tw; 2 School of Medicine, College of Medicine, Fu Jen Catholic University, New Taipei City 242, Taiwan; 3 Graduate Institute of Clinical Medicine, College of Medicine, National Taiwan University, Taipei 10002, Taiwan; 4 Department of Ophthalmology, National Taiwan University Hospital, Taipei 10002, Taiwan; chienchiasu@ntu.edu.tw (C.-C.S.); liuyaolin@ntuh.gov.tw (Y.-L.L.); F630@ktgh.com.tw (I.-J.T.)

**Keywords:** strabismus, ADHD, esotropia

## Abstract

Strabismus is associated with amblyopia and a lower quality of life. Attention-deficit hyperactivity disorder (ADHD) is common among children and adolescents, and influences their academic, vocational, and social life. Previous studies have suggested an association between strabismus and ADHD. Using data from the Taiwan National Health Insurance Research Database between 2000 and 2010, we performed a large-scale cohort study comparing the incidence, risk factors, and severity of ADHD in children with and without strabismus. A total of 2049 patients <18 years old with newly diagnosed strabismus (esotropia: 404; exotropia: 1645) were identified, and 8196 age- and sex-matched controls without strabismus were also included. After an average of 6.5 ± 2.9 years of follow-up, the incidence of ADHD per 1000 person-years was 5.39 in the strabismus group (esotropia: 9.93; exotropia: 4.11) and 3.23 in the control group. The cumulative incidence of ADHD was significantly greater in the esotropia (hazard ratio [HR]: 2.04; 95% confidence interval [CI]:1.36–3.06; *p* = 0.0007) and exotropia groups (HR: 1.44; 95% CI: 1.03–2.03; *p* = 0.038) than in the controls. Patients with strabismus had more comorbidities than those without (*p* < 0.05). In summary, this large-scale study found a higher cumulative incidence of ADHD in patients with strabismus, especially in those with esotropia.

## 1. Introduction

Strabismus is a common ocular alignment disorder that affects 1–5% of children worldwide [1,2,3,4]. Exotropia, an outward deviation, is more common in Asian populations, while esotropia, an inward deviation, predominates among children in the West [5]. In addition to eye deviation, strabismus may lead to amblyopia or lack of stereopsis [6,7], and some patients require surgical treatment to improve cosmetic appearance and binocular vision. Strabismus may also lead to low self-esteem and impair learning performance [8]. In recent studies, strabismus has also been found to be related to a reduced quality of life in both patients and their parents [9], lower health-related quality of life in children and adolescents [10], and higher rates of mental health problems or psychiatric disease, especially in patients with intermittent exotropia [11]. Using functional magnetic resonance imaging (fMRI), neuroimaging studies have revealed changes in the activities in the central nervous system in subjects with amblyopia [12] and strabismus [13].

Attention-deficit hyperactivity disorder (ADHD) is a common psychiatric disorder among children and adolescents. Overall, the pooled estimated prevalence is 7.2% in school and community populations [14]. ADHD is a multifactorial disease, and the diagnosis requires long-term follow-up and complete evaluation by psychiatrists. The clinical presentation of ADHD includes inattentiveness, hyperactivity, impulsivity, and cognitive deficits [15], and the symptoms are related to poor performance in reading and mathematical skills and lower grades [16,17]. Early complications of ADHD include anxiety and learning disorders, but the psychosocial consequences of ADHD can be lifelong [18]. Patients diagnosed with ADHD may suffer from long-lasting difficulties in academic, vocational, and social life and require continuous pharmacologic or psychosocial treatment [19,20,21].

In the past decade, strabismus has been found to be related to mental health problems in both children and adults [11,22,23,24,25]. In a Western study, patients with exotropia—especially intermittent type—in particular were more prone to develop mental health problems than non-strabismus controls, and the top five mental health disorders related to strabismus were depression, ADHD, adjustment disorder, illegal drug use, and alcoholism [11]. Surgery for strabismus, regardless of age at surgery or success, did not change the development of mental illness [25]. Convergence insufficiency has been associated with a higher incidence of ADHD [26]. Despite increasing studies suggesting an association between strabismus and mental illness, including ADHD, most studies were limited by a small sample size and a lack of a comparative follow-up design, preventing researchers from reaching a definite conclusion regarding the correlation between strabismus and ADHD.

The National Health Insurance (NHI) program is a mandatory health insurance program that covers 99% of the 23 million residents in Taiwan. The Taiwan National Health Insurance Research Database (NHIRD) contains the medical records of inpatients, outpatients, emergency care, surgery, and prescription records. We utilized the Taiwan NHIRD to conduct a large cohort study to compare the incidence, risk, and severity of ADHD between children with and without strabismus in Taiwan from 2000 to 2010.

## 2. Materials and Methods

This retrospective population-based cohort study used data from the Taiwan NHIRD. In this study, we included patients aged ≤18 years with exotropia or esotropia between 2000 and 2010. Patients with a previous diagnosis of ADHD were excluded. For each included insurant, we recruited four sex-and age-matched controls without a diagnosis of exotropia or esotropia. We also collected data regarding comorbidities such as autism, Tourette syndrome, depression, delayed development, intellectual disability, lower respiratory tract infection, and epilepsy.

**Figure 1:**
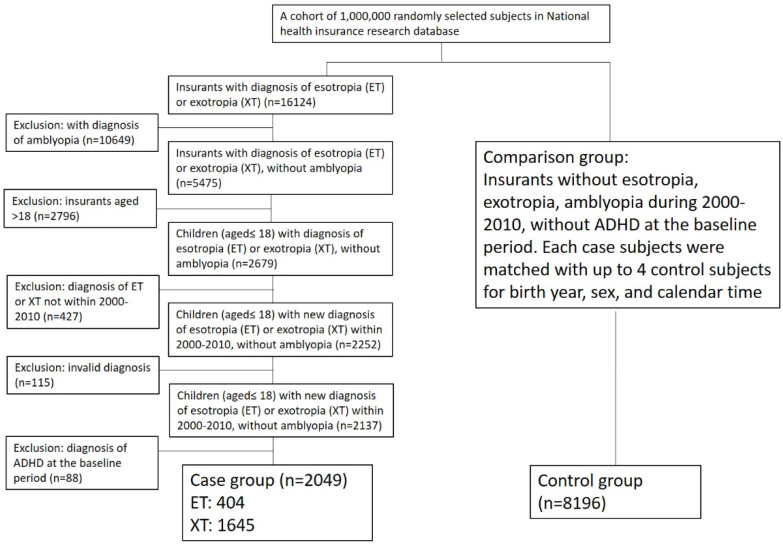
Overview of the enrollment of the study participants from the National Health Insurance Research Database.

We used the Longitudinal Health Insurance Database of the NHIRD. This database randomly selected 1,000,000 patients from registered insurants from the NHI program. We defined esotropia in this study as International Classification of Diseases, Ninth Revision, Clinical Modification (ICD-9-CM) codes: 378.0, 378.21, 378.22, 378.35, 378.41, 378.84, and 378.85 and exotropia as ICD-9-CM codes: 378.1, 378.23, 378.24, and 378.42. To avoid errors during data collection and to confirm the accuracy of the diagnosis, only those with the same diagnosis (esotropia or exotropia) occurring at least three times were included. Patients with a diagnosis of both esotropia and exotropia, even when these occurred at different times, were excluded. To rule out the effect of suboptimal visual acuity on the mental development of children, we excluded individuals with a diagnosis of amblyopia (ICD-9CM codes: 368.00, 368.01, 368.02, or 368.03). Since this study was designed to determine the incidence per year of ADHD, patients with a diagnosis of ADHD (ICD-9CM codes: 314.00, 314.01, 314.1, 314.2, 314.8, or 314.9) at baseline were also excluded (Figure 1). Age- and sex-matched insurants without exotropia, esotropia, and amblyopia served as the control group. In those who developed ADHD during follow-up, we recorded the age at first diagnosis of ADHD to analyze the differences in age at disease development between insurants with and without strabismus.

The primary outcome was the development of ADHD. Diagnosis of ADHD was based on clinical evaluation (DMS-IV-TR) by psychiatrists (ICD-9CM code: 314). All patients were followed-up until the development of ADHD or until they dropped out from the study. The secondary outcome was medical usage in patients with ADHD. Medication usage duration and dosage may reflect the severity of ADHD. Thus, for disease severity analysis, we collected the medical records of these patients, including duration and age at the start of medical treatment.

### Statistical Analysis

We used the SAS statistical package for Windows, version 9.1, (SAS Institute Inc., Cary, NC, USA) for the study analysis. The Pearson X^2^ test was performed to examine the study and control cohorts in terms of demographic data; The Wilcoxon rank sum test was applied for mean value analysis; and the Kaplan–Meier method was used for survival plot analysis for ADHD development in patients with esotropia or exotropia and control groups. The Cox proportional hazard model was used for multivariate analysis. *p* < 0.05 was considered statistically significant.

## 3. Results

In total, 404 patients with esotropia, 1645 with exotropia, and 8196 age- and sex-matched controls were included in this study. A flowchart of the study is presented in Figure 1. The mean age of patients with strabismus was 9.2 ± 4.2 years (esotropia: 6.9 ± 4.7 years; exotropia: 9.8 ± 3.8 years). The mean age of the control group was 9.2 ± 4.2 years. The mean follow-up time was 7.2 ± 3.0 years in esotropia patients, 6.4 ± 2.9 years in exotropia patients, and 6.5 ± 2.9 years in the control group (Table 1). During the observational period, ADHD was diagnosed in 29 patients with esotropia, 43 patients with exotropia, and 173 patients in the control group. In patients with strabismus, the incidence rate of ADHD per 1000 person-years was 5.39 (esotropia: 9.93; exotropia: 4.11) compared with 3.23 in the control group (Table 1). Due to the difficulties in diagnosing ADHD in patients under five years of age, we further compared the incidence of ADHD between the strabismus and control groups under five years of age. The results showed that only five patients with strabismus (four with esotropia and one with exotropia) and seven patients without strabismus were diagnosed with ADHD under five years of age. There was no significant difference in ADHD incidence between the strabismus and non-strabismus group under five years of age (*p* = 0.07).

An analysis of comorbidities found that patients with strabismus had more comorbidities than those in the control group. Comorbidities included autism spectrum disorder, depression, delayed development, intellectual disability, encopresis, prematurity, perinatal infection, lower respiratory tract infection, paralysis, headaches, epilepsy, rheumatic disease, chronic pulmonary disease, asthma, allergic rhinitis, allergic conjunctivitis, and deficiency anemia (All *p* < 0.05; Table 1).

**Figure 2:**
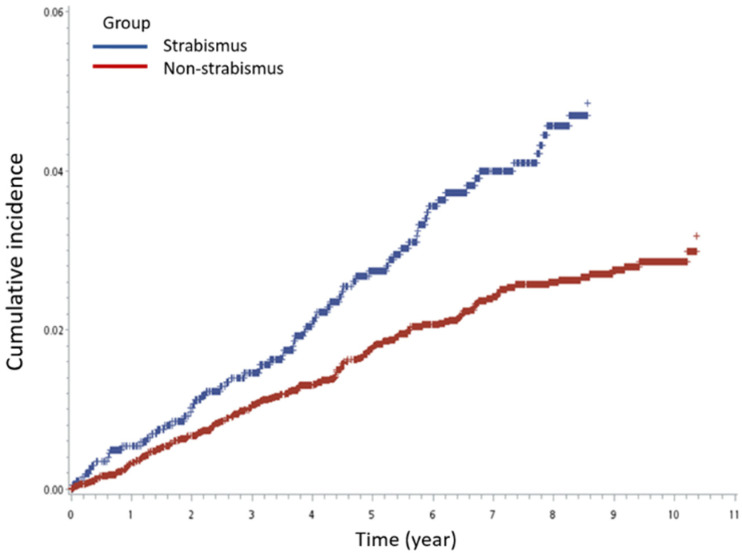
Kaplan–Meier survival analysis shows the cumulative incidence of attention-deficit hyperactivity disorder over 10 years in the strabismus and non-strabismus control groups.

**Figure 3:**
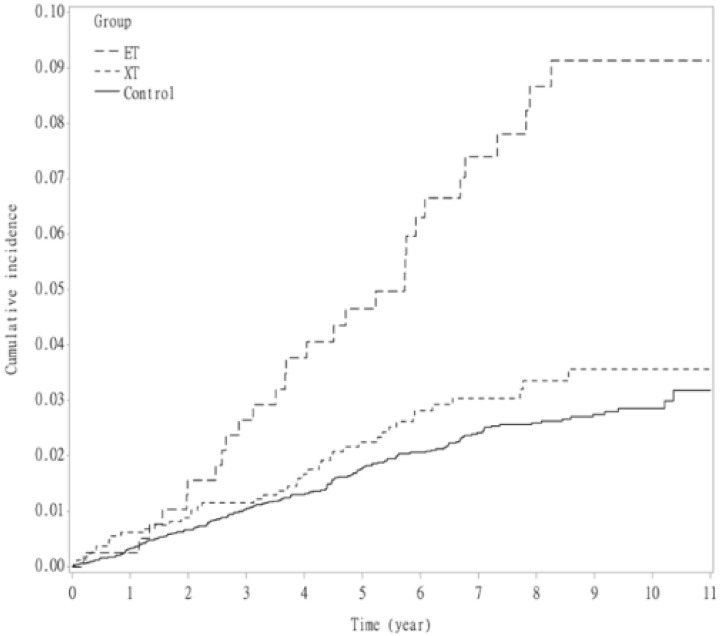
Kaplan–Meier survival analysis shows the cumulative incidence of attention-deficit hyperactivity disorder over 10 years in patients with esotropia, exotropia, and those without strabismus. ET, esotropia; XT, exotropia.

The results of the Cox regression analysis are shown in Table 2. Patients with strabismus had a significantly higher incidence of ADHD development (adjusted hazard ratio(aHR): 1.64; 95% confidence interval (CI): 1.23–2.17; *p* < 0.001) after adjustment for age, sex, autism, delayed development, intellectual disability, lower respiratory tract infection, paralysis, and prematurity (Figure 2). The incidence of ADHD was significantly higher in the esotropia group than in the control group (aHR: 2.04; 95% CI: 1.36–3.06; *p* < 0.001). Patients with exotropia did not have a higher incidence of ADHD than patients in the control group in crude comparison, but there was a significant difference in multivariate analysis (aHR: 1.44; 95% CI: 1.03–2.03; *p* = 0.038; Figure 3).

The average age of onset of ADHD was 9.35 ± 3.06 (range, 3.79–17.91) years in the strabismus group (esotropia, 8.53 ± 2.92 (3.79–16.67), and exotropia, 9.90 ± 3.06 (4.44–17.91)) and 9.34 ± 2.85 (2.73–16.06) years in the control group. There was no significant difference between the groups.

To evaluate the severity of ADHD, we further analyzed the use of medical treatment in patients with ADHD. In patients with strabismus (both esotropia and exotropia), 41 (56.9%) were under medical treatment, while 31 (43.1%) were not. In the non-strabismus group, 106 (61.3%) patients were under medical treatment, and 67 (38.7%) were not; there were no significant differences between the strabismus and control groups (*p* = 0.53). Among those receiving medical treatment for ADHD, the median duration of treatment was 4.43 months in the strabismus group and 3.54 months in the control group, with no significant difference between groups (*p* = 0.96; data not shown).

## 4. Discussion

This large-scale cohort study investigated the incidence, risk, and severity of ADHD in children with and without strabismus. Our results indicated that patients under 18 years of age with horizontal strabismus, and especially esotropia, had a higher incidence of ADHD in a 10-year follow-up. Furthermore, patients with strabismus had more comorbidities than those in the control groups, including mental disorders, developmental disorders, and neurological and respiratory disorders.

The higher incidence of ADHD in patients with strabismus found in our study is in line with previous reports. A cross-sectional study by DeCarlo et al. revealed a higher prevalence of ADHD in children with vision impairment [27]. Garnet et al. found a three-fold increase in the incidence of ADHD among patients with convergence insufficiency compared with the general population in a retrospective study [26]. In a large-scale cohort study by Reimelt et al., strabismus and refractive error were linked to ADHD [28]. In another large-scale cross-sectional telephone survey in the United States [29], children with eye problems had a higher prevalence of ADHD (15.6%) than those with normal vision (8.3%). Our study had several advantages compared with previous studies, including a larger sample size, more specific diagnosis, and evaluation of the age of disease onset and cumulative incidence; further, we performed specific analyses of medication use. We also found that the type of strabismus may influence the development of ADHD, with esotropia having a higher relative risk for the development of ADHD.

Previous studies [11,26] found a stronger association of exotropia with mental illness and ADHD, which is contrary to the results of our study. The hazard ratio for the incidence of ADHD was 2.04 in the esotropia group and 1.44 in the exotropia group. This difference may be due to the demographic distribution of the strabismus type in Eastern and Western countries. For example, a study conducted in Singapore and China found that exotropia was more prevalent than esotropia in Asian countries, and intermittent basic type was the most common [30,31]. Unlike esotropia, intermittent exotropia is usually characterized by an older age of onset and preservation of stereovision while the eyes are aligned [1,32,33]. In our study, the mean age at diagnosis of esotropia was lower than that of exotropia (*p* < 0.005), and more than half (57.7%) of the children with esotropia were diagnosed under the age of 7, while the most common (43.8%) age distribution was between 8 and 12 years in our exotropia subgroup. Earlier disorders of the eye, including strabismus and cataract, may have a more profound effect on neurodevelopment in children [34]. Besides this, some predisposing factors for ADHD, such as maternal smoking, have also been proven to be risk factors for esotropia, but not of exotropia [35]. Early esotropia can also lead to anisometropia and/or amblyopia [36]. In our previous study, we identified amblyopia as an independent risk factor for the development of ADHD [37]. Although we excluded patients with a diagnosis of amblyopia in this study, children with esotropia, who have a higher chance of developing amblyopia, were found to have a higher risk of developing ADHD.

Although the causal relationship between strabismus and ADHD is not fully understood, neuroimaging studies revealed structural brain alterations both in strabismus and ADHD patients [13,38,39,40,41,42,43,44]. In a resting-state functional MRI study of patients with comitant strabismus, increased regional homogeneity was found in the right inferior temporal cortex, fusiform gyrus, cerebellum anterior lobe, right lingual gyrus, and bilateral cingulate gyrus [13]. Using resting-state functional MRI, Castellanos et al. suggested cingulate–precuneus interactions as a new locus of dysfunction in ADHD [40]. Another study suggested that the entire cingulate cortex is engaged in the structure/function abnormalities found in ADHD [41]. Furthermore, adults with strabismic amblyopia had white matter tract alterations in the anterior frontal corpus callosum, right vertical occipital fasciculus, left inferior longitudinal fasciculus, and left optic radiation [39]. In infantile esotropia, asymmetrical cortical connections via the corpus callosum were revealed by diffusion tensor imaging [42]. In a neuroimaging study of ADHD subjects, macrostructural abnormalities, microstructural alterations, and anomalous growth trajectories in the corpus callosum were also noted compared with healthy controls [43,44]. Although there was some overlap of the abnormal brain regions found in strabismus and ADHD patients, further longitudinal studies are necessary to elucidate the relationships between strabismus, ADHD, and neural structural alterations.

The limitation of the present study mostly originates from the nature of the NHIRD [45]. For example, coding errors, missing data due to failure to seek medical treatment for ADHD or strabismus, and lack of inclusion of records of self-pay healthcare and out-of-pocket payments may be confounding factors for the NHIRD study. In addition, the severity of diseases and environmental factors could not be revealed simply by the ICD-9-CM codes. Since ADHD is a multifactorial disease, factors such as the socioeconomic status of parents and the learning and residential environment may influence its incidence. The NHIRD still requires more comprehensive validation, and the results should be interpreted carefully. Although the primary outcome of the current study might not be significantly affected, the exclusion of amblyopia may decrease the case number of esotropia, and the rate of autism or prematurity in co-morbidity analysis. Excluding patients with diagnoses of both esotropia and exotropia may have also excluded patients with surgery and over-correction, which represent a cohort with a more advanced strabismus. Nevertheless, our results were derived from the analysis of a nationally representative large cohort and should be of scientific value.

## 5. Conclusions

Our study found a higher cumulative incidence of ADHD in patients with strabismus in a large nationwide database with an average of 6.5 ± 2.9 years of follow-up. The relative risk of ADHD was higher in children with esotropia than in those with exotropia and without strabismus. Further neuroimaging or field survey studies are required to understand the underlying pathomechanisms.

## Figures and Tables

**Table 1:** Characteristics of patients with esotropia, exotropia (case group) and the non-strabismus subjects.

Variable	Total	Strabismus Group	Esotropia	Exotropia	Non-Strabismus Group	*p*-Value *
	n = 44330	n = 2049	n = 404	n = 1645	n = 8196	
Age, no. (%)						
≤7	3197 (31.2)	650 (31.7)	233 (57.7)	417 (25.3)	2547 (31.1)	0.7168
7– ≤ 12	4180 (40.8)	820 (40.0)	100 (24.8)	720 (43.8)	3360 (41.0)	
12– ≤ 18	2868 (28.0)	579 (28.3)	71 (17.6)	508 (30.9)	2289 (27.9)	
Mean (SD)	9.2 (4.2)	9.2 (4.2)	6.9 (4.7)	9.8 (3.8)	9.2 (4.2)	0.9987
Female	5440 (53.1)	1088 (53.1)	219 (54.2)	869 (52.8)	4352 (53.1)	1.0000
Duration of follow-up (year)						
Mean (SD)	6.5 (2.9)	6.5 (2.9)	7.2 (3.0)	6.4 (2.9)	6.5 (2.9)	0.7877
Median (interquartile range)	6.9 (4.4, 9.1)	6.9 (4.3, 9.1)	8.1 (5.1, 9.8)	6.7 (4.2, 8.9)	6.9 (4.4, 9.1)	
Comorbiditities **, no. (%)						
Autism spectrum disorder	7 (0.1)	7 (0.3)	1 (0.2)	6 (0.4)	0 (0.0)	<0.0001
Tourette syndrome (Tics)	3 (0.0)	2 (0.1)	0 (0.0)	2 (0.1)	1 (0.0)	0.1040
oppositional defiant disorder	0 (0.0)	0 (0.0)	0 (0.0)	0 (0.0)	0 (0.0)	
depression	4 (0.0)	3 (0.1)	1 (0.2)	2 (0.1)	1 (0.0)	0.0272
Delayed development	48 (0.5)	37 (1.8)	12 (3.0)	25 (1.5)	11 (0.1)	<0.0001
Intellectual disability	10 (0.1)	7 (0.3)	1 (0.2)	6 (0.4)	3 (0.0)	0.0009
Stuttering	0 (0.0)	0 (0.0)	0 (0.0)	0 (0.0)	1 (0.0)	
Obsessive compulsive disorder	1 (0.0)	0 (0.0)	0 (0.0)	0 (0.0)	1 (0.0)	0.6171
Conduct disorder	0 (0.0)	0 (0.0)	0 (0.0)	0 (0.0)	1 (0.0)	
Encopresis	6 (0.1)	4 (0.2)	0 (0.0)	4 (0.2)	2 (0.0)	0.0043
Feeding and eating disorder	0 (0.0)	0 (0.0)	0 (0.0)	0 (0.0)	0 (0.0)	
Prematurity, low birth weight	7 (0.1)	6 (0.3)	2 (0.5)	4 (0.2)	1 (0.0)	0.0004
Perinatal infection	5 (0.0)	5 (0.2)	5 (1.2)	0 (0.0)	0 (0.0)	0.0003
Fetal and newborn respiratory condition	1 (0.0)	1 (0.0)	0 (0.0)	1 (0.1)	0 (0.0)	0.2000
Birth condition	2 (0.0)	1 (0.0)	0 (0.0)	1 (0.1)	1 (0.0)	0.3600
Birth trauma	1 (0.0)	0 (0.0)	0 (0.0)	0 (0.0)	1 (0.0)	1.0000
Intrauterine hypoxia and birth asphyxia	1 (0.0)	1 (0.0)	1 (0.2)	0 (0.0)	0 (0.0)	0.2000
Lower respiratory tract infection	1846 (18.0)	597 (29.1)	159 (39.4)	438 (26.6)	1249 (15.2)	<0.0001
Paralysis	29 (0.3)	26 (1.3)	4 (1.0)	22 (1.3)	3 (0.0)	<0.0001
Headaches	74 (0.7)	40 (2.0)	8 (2.0)	32 (1.9)	34 (0.4)	<0.0001
Epilepsy	28 (0.3)	16 (0.8)	0 (0.0)	16 (1.0)	12 (0.1)	<0.0001
Rheumatic disease	2 (0.0)	2 (0.1)	0 (0.0)	2 (0.1)	0 (0.0)	0.0400
Chronic pulmonary disease	376 (3.7)	112 (5.5)	23 (5.7)	89 (5.4)	264 (3.2)	<0.0001
asthma	289 (2.8)	90 (4.4)	18 (4.5)	72 (4.4)	199 (2.4)	<0.0001
allergic rhinitis	606 (5.9)	245 (12.0)	49 (12.1)	196 (11.9)	361 (4.4)	<0.0001
allergic conjunctivitis	191 (1.9)	91 (4.4)	8 (2.0)	83 (5.0)	100 (1.2)	<0.0001
Deficiency anemias	6 (0.1)	4 (0.2)	0 (0.0)	4 (0.2)	2 (0.0)	0.0169

* *p-values* for case group (column C) and control group (column F) were calculated by Chi-square test, Fisher’s exact test, Wilcoxon rank sum tests or Brown–Mood test. ** Defined 1 year before the index date.

**Table 2:** Hazard ratios for attention deficit hyperactivity disorder (ADHD) in strabismus patients, compared to the non-strabismus group.

	ADHD Events	PY	Incidence ^†^	Hazard Ratio (95% C.I.)	
				Crude	Adjusted
Comparison group	173	53615	3.23	1 (reference)	1 (reference)
All patients	72	13370	5.39	1.67 (1.27, 2.20) **	1.64 (1.23, 2.17) **
Patients with ET	29	2920	9.93	3.14 (2.12, 4.65) **	2.04 (1.36, 3.06) **
Patients with XT	43	10450	4.11	1.27 (0.91, 1.77)	1.44 (1.03, 2.03) *

PY, person-year; ET, esotropia; XT, exotropia. ^†^ per 1000 person-years. * *p* < 0.01; ** *p* < 0.0001. Models adjusted for age, sex, autism, delayed development, intellectual disability, lower respiratory tract infection, paralysis, and prematurity.

## Data Availability

The data of this study are available from the National Health Insurance Research Database (NHIRD) in Taiwan, but were used under license for the current study and thus are not publicly available.
